# The Usefulness of Confusion, Urea, Respiratory Rate, and Shock Index or Adjusted Shock Index Criteria in Predicting Combined Mortality and/or ICU Admission Compared to CURB-65 in Community-Acquired Pneumonia

**DOI:** 10.1155/2013/590407

**Published:** 2013-08-20

**Authors:** James P. Curtain, Prasanna Sankaran, Ajay V. Kamath, Phyo K. Myint

**Affiliations:** ^1^Addenbrooke's University Hospital, Cambridge, Cambridgeshire CB2 0QQ, UK; ^2^School of Medicine & Dentistry, Division of Applied Health Sciences, C/o Room 4013, Polwarth Building, Foresterhill, Aberdeen AB25 2ZD, UK; ^3^Department of Respiratory Medicine, Norfolk and Norwich University Hospital, Norwich, Norfolk NR4 7UY, UK; ^4^School of Medicine, Health Policy and Practice, University of East Anglia, Norwich, Norfolk NR4 7TJ, UK; ^5^School of Medicine & Dentistry, University of Aberdeen, Foresterhill, Aberdeen AB25 2ZD, UK

## Abstract

*Background and Objectives*. The study aims to assess the usefulness of age-independent criteria CURSI and temperature adjusted CURSI (CURASI) compared to CURB-65 in predicting community-acquired pneumonia (CAP) mortality. The criteria, CRSI and CRASI, were adapted for use in primary care and compared to CRB-65. *Methods*. A retrospective analysis of a prospectively identified cohort of community-acquired pneumonia inpatients was conducted. Outcomes were (1) mortality and (2) mortality and/or ICU admission within six weeks. *Results*. 95 patients (median age = 61 years) were included. All three criteria had similar sensitivity in predicting mortality alone, with CURB-65 having slightly higher specificity. When predicting mortality and/or intensive care admission, CURSI/CURASI showed higher sensitivity and slightly lower specificity. CRSI and CRASI had higher sensitivity and lower specificity when compared with CRB-65 for predicting both primary and secondary outcomes. Results for both analyses had *P* values >0.05. *Conclusions*. In a cohort of younger patients CURSI and adjusted CURSI perform at least as well as CURB-65, with a similar trend for CRSI and adjusted CRSI compared to CRB-65. Further studies are needed in different age groups and in primary and secondary care settings.

## 1. Introduction

Community-acquired pneumonia (CAP) is a leading cause of hospital admission, patient death, and a significant burden on health service resources in the UK [1]. Mortality rates are highest in patients considered to have severe pneumonia [2–5]. Ultimate mortality rates for patients requiring ICU admission have been reported at over 40% [6, 7]. Early recognition of severely ill patients is important to predict those with the worst prognosis, and a dependable method of assessing pneumonia severity may refine initial management of these patients by helping clinicians decide whether close monitoring and aggressive treatment are required. Correctly identifying severe community-acquired pneumonia cases will also reduce the inappropriate use of intravenous antibiotics, thereby reducing the risk of antibiotic-associated colitis, which is an important issue for health service utilisation, mortality, and morbidity [8]. The CURB-65 severity index, recommended by the British Thoracic Society [9], compares similarly with other severity scores [10] and is a readily applied tool that can be used to guide site of care and degree of medical intervention. Recent comprehensive reviews [10, 11] have drawn attention to the limitations of current established community-acquired pneumonia severity assessment criteria. 

CURB-65 criteria are based on; confusion; urea > 7 mmol/L; respiratory rate ≥ 30/min; systolic (<90 mmHg) or diastolic (≤60 mmHg) blood pressure; and age ≥65 years, scoring one mark each [5]. Patients with scores of 3 or more are classed as having severe pneumonia. While younger and older patients alike have been shown to mount similar immunoresponses to pneumonia infection [12], concerns over the use of age-dependent scoring systems have been raised given the variability of presentation between different age groups [13]. The potential for misleadingly low scores in patients younger than 65 years has been declared [14, 15]. The inclusion of chronological age in severity assessments may also lead to false positive results amongst some older patients while, conversely, systolic hypertension with increasing age may lead to false negatives in older patients. It has also been suggested that low diastolic blood pressure (≤60 mmHg) is not predictive of mortality in older patients [16, 17] and may be due to reductions in mean diastolic blood pressure with physiological ageing. Diastolic blood pressure criteria may therefore not have the same implication for older patients as for younger patients, further complicating its use as part of CURB-65 severity scoring. Cardiovascular shock in community-acquired pneumonia has previously been identified as an independent prognostic factor for mortality [18]. We have previously reported that using the shock index (SI), the ratio of heart rate to systolic blood pressure, in place of age may have future potential use based on retrospectively collected audit data [19] as part of a scoring criteria combined with confusion, urea, and respiratory rate (CURSI/CURASI). CURSI and CURASI criteria showed comparable usefulness to CURB-65 in predicting mortality in a cohort of older patients with a median age of 76 years (*n* = 190) [20].

The CURB-65 derivation study [5] also presented the CRB-65 criteria which is adapted for use in primary care. The exclusion of urea as a marker of severity negates the need for any biochemical testing, and the patient's score is calculated solely on clinical measurements which can be recorded by a general practitioner before deciding on whether to admit a patient to hospital or maintain care within the community. Such decisions have important implications for both the patient, the healthcare professionals looking after them, and the resources available to both. CRB-65 has shown similar effectiveness to CURB-65 in the hospital setting, and the measurement of urea may not offer any further predictive ability to CURB-65 [4, 21–24]. A recent meta-analysis of CRB-65 [25] reports howerver that it overpredicts the probability of mortality in the community setting and should be used with caution.

The aim of this study was to assess the usefulness of CURSI and CURASI compared to CURB-65 in predicting community-acquired pneumonia (CAP) severity in a relatively young patient cohort. CRB-65 was also compared to CRSI and CRASI scores in a separate analysis.

## 2. Methods

Unselected adults admitted to a respiratory team in a large acute general hospital with community-acquired pneumonia were included in the study. CAP was defined as the presence of new shadowing on admission chest X-ray and clinical features consistent with pneumonia (e.g., cough, sputum expectoration, shortness of breath, and pleuritic chest pain with or without fever). Only patients in whom CAP was the main reason for admission were included. Any patients in whom there was an expected terminal event or who developed hospital-acquired pneumonia were excluded from the study. Patients were followed up in the respiratory medical clinic six weeks following admission. Data were collected prospectively over a 14-month period (April 1999–June 2000), as has been previously reported [3]. Hospital guidelines based on a previous British Thoracic Society study [21] were in use throughout this time period and used in guiding patient care. Analysis was conducted retrospectively, using mortality at 6 weeks and mortality and/or ICU admission at 6 weeks as outcomes. Data was collected for 96 patients in total, with one patient being excluded from the CURB-65/CURSI/CURASI analysis (*n* = 95) and a different patient being excluded from the CRB-65/CRSI/CRASI analysis (*n* = 95) due to missing values.

In this study, CURSI scores were derived from confusion; urea > 7 mmol/L; respiratory rate ≥ 30/min; and a shock index ratio > 1.0, scoring one mark each. CURSI was also applied with an adjusted shock index score (CURASI) where 10 points were deducted from the heart rate for every 1.0°C increase in the patient's temperature above 37.0°C [20]. This adjustment was to account for the physiological increase in heart rate with rising temperature. CURSI and CURASI scores of 2 or more were classed as severe pneumonia. Except for the exclusion of urea as a marker of severity, CRB-65 and CRSI/CRASI criteria were calculated using the same variables and cutoff points. CRB-65 scores of 3 or more and CRSI/CRASI scores of 2 or more were classed as severe.

Five patients had one data variable missing from CURB-65, CURSI, and CURASI severity scoring, with one patient being excluded from the study due to a borderline score and allocation to the non-severe group which may have been switched to the severe group depending on the missing urea value. However, due to the results of the remaining four patients' recorded data (non-severe patients all scored 0 points, *n* = 3; severe patient scored 2 points [CURSI/CURASI] and 3 points [CURB-65], *n* = 1) these patients were confirmed in the groups they were originally allocated, irrespective of any absent values. One patient was excluded from CRB-65, CRSI, and CRASI analyses due to a missing value for confusion status, which gave the patient a borderline non-severe CRB-65 score of 2 and CRSI/CRASI scores of 1. The addition of a positive confusion value may have resulted in an allocation to the severe group, and the patient was therefore excluded. The adjustment of shock index to calculate the CURASI and CRASI scores resulted in different values for the shock index in forty-seven patients. One patient was reclassified from the severe to non-severe group in the CRSI/CRASI analysis. None were reclassified in the CURSI/CURASI analysis.

The sensitivities, specificities, and positive and negative predictive values were calculated for each of the criteria. Comparisons between the sensitivity and specificity results for both CURSI/CURASI and CURB-65 and for CRSI/CRASI and CRB-65 were performed using the Wilcoxon Signed-Rank Test. Statistical significance was taken as a *P* value of ≤0.05. SPSS 18.0 (IBM, NY, USA) statistical package was used to analyse the data. 

The study data was collected as part of an internal audit cycle, and so local ethical committee approval was not required.

## 3. Results

95 patients (54 males and 41 females) were eligible to be included in the current report. No patient with missing data for parameters required for severity scoring was included in the analysis, unless their remaining recorded values confirmed their allocation to either the non-severe or severe group. Patients were aged 17–96 years (median age 61 years, mean age 58.8 years). Eight patients died (aged 55–80 years) during the study. Ten patients were admitted to ICU, six of whom died. Three of the four ICU survivors were aged less than 65 years. One of these patients was excluded from the CURSI/CURASI/CURB-65 analysis due to missing urea values but was included in the CRSI/CRASI/CRB-65 analysis. Therefore, eleven patients either died or were admitted to ITU in the CURSI/CURASI/CURB-65 analysis, while there were twelve such patients in the CRSI/CRASI/CRB-65 analysis. 

**Figure 1:**
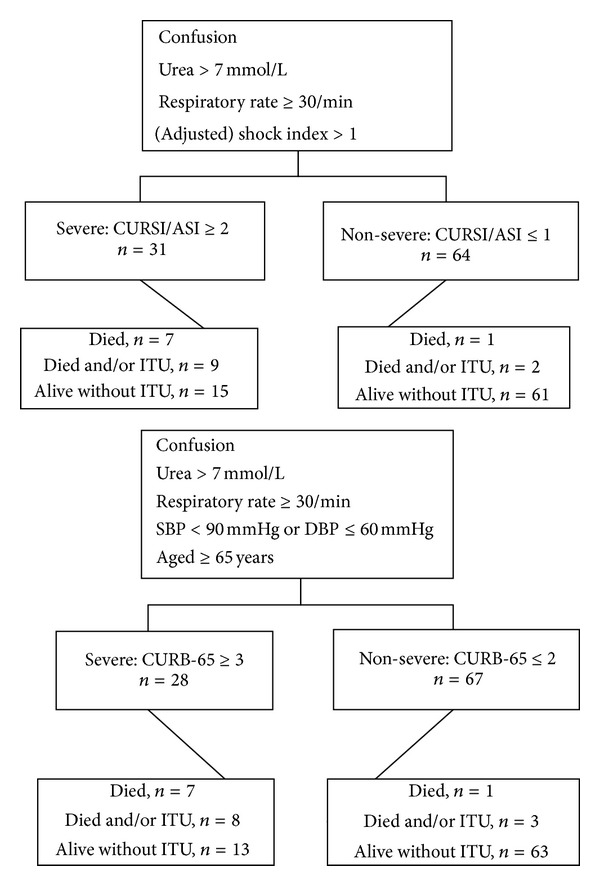
Severity and outcomes according to CURSI, CURASI, and CURB-65 groups, *n* = 95.

Figure 1 presents the severity groups to which patients were allocated according to CURSI/CURASI/CURB-65 and their outcomes. CURSI and CURASI performed similarly, identifying 31 (32%) of 95 patients as having severe pneumonia. 7 of whom died. CURSI and CURASI classed 9 of the patients who either died and/or were admitted to ITU as severe. CURB-65 classed 28 (29%) of the cases as having severe pneumonia. 7 of these patients died. CURB-65 classed 8 patients who either died and/or were admitted to ITU as severe.

**Figure 2:**
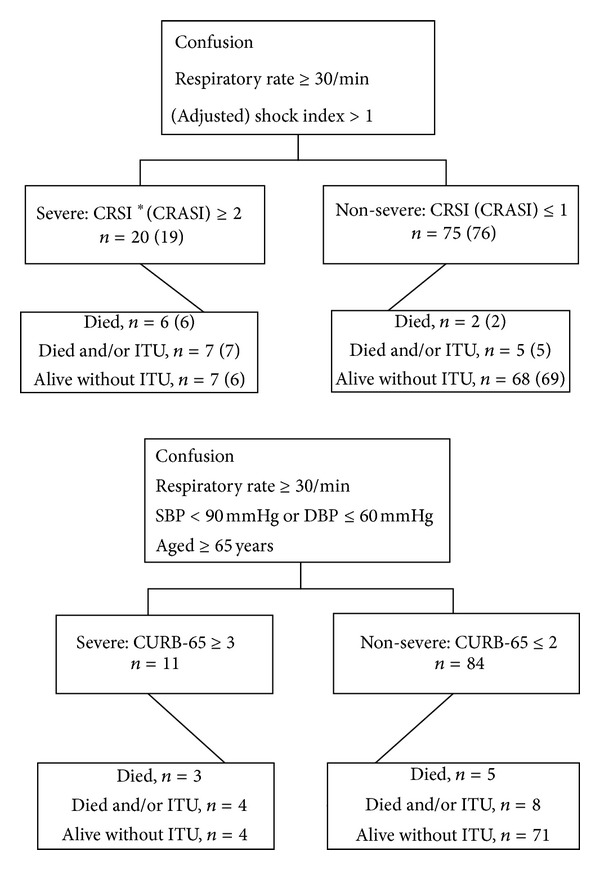
Severity and outcomes according to CRSI, CRASI, and CRB-65 groups, *n* = 95. *CRASI figures are detailed in ().

Figure 2 presents the severity groups to which patients were allocated according to CRSI/CRASI/CRB-65 and their outcomes. CRSI classed 20 (21%) of 95 patients as severe. CRASI classed 19 (20%) patients as severe. CRSI and CRASI classed 6 of the 8 patients who died as severe and classed 7 of the patients who either died and/or were admitted to ITU as severe. CRB-65 classed 11 (12%) patients as severe, of whom 3 died and a fourth patient was admitted to ITU. 

Table 1 presents the cohort sample characteristics, including for age, gender, confusion, urea levels, respiratory rate, blood pressure, heart rate, and temperature. Median (ranges)/mean (±sd) values and numbers (percentages) are detailed.

Table 2 presents sensitivity, specificity, and positive and negative predictive values of CURB-65, CURSI, and CURASI calculated for mortality and for mortality and/or ICU admission. All three severity indices compared similarly for sensitivity (87.5%, CI 46.7–99.3, and *P* = 0.18) for mortality alone, with CURB-65 having 3.4% higher specificity (75.8%, CI 65.3–84.1) than CURSI/CURASI (72.4%, CI 61.6–81.1, and *P* = 1.00). When predicting for mortality and/or intensive care admission, the results suggest that CURSI/CURASI showed 9.1% higher sensitivity (81.8%, CI 47.7–96.8) compared to CURB-65 (72.7%, CI 39.3–92.6, and *P* = 0.31) with a slightly lower (2.4%) specificity than CURB-65 (*P* = 0.31). Results did not reach a significance level of *P* < 0.05, as will be discussed in the next section.

Table 3 presents sensitivity, specificity, and positive and negative predictive values for CRB-65, CRSI, and CRASI with regard to mortality and to mortality and/or ICU admission. The results suggest the higher sensitivity of CRSI (75%, CI 35.6–95.5, and *P* = 0.15) and CRASI (75%, CI 35.6–95.5, and *P* = 0.25) when compared with CRB-65 (50%, CI 17.4–82.5) for predicting mortality, with a higher specificity of CRB-65 (91.9%, CI 83.6–96.4) than CRSI (83.9%, CI 74.1–90.6, and *P* = 0.15) and CRASI (85.0%, CI 75.4–91.5, and *P* = 0.15). In predicting mortality and/or intensive care admission the results followed the same trend by suggesting the higher sensitivity of CRSI (58.3%, CI 28.6–83.5, and *P* = 0.18) and CRASI (58.3%, CI 28.6–83.5, and *P* = 0.41) to CRB-65 (33.3%, CI 11.3–64.6) and a higher specificity with CRB-65 (91.6%, CI 82.8–96.2) when compared with CRSI (84.3%, CI 74.3–91.1, and *P* = 0.08) and CRASI (85.5%, CI 75.7–91.9, and *P* = 0.15). 

## 4. Discussion

The study has some limitations, the foremost of which is the small sample size. Statistically the comparisons between the criteria did not reach significance (*P* ≤ 0.05), which may be attributable to this sample size. However without a statistical difference between the groups, the indication is that both CURSI/CURASI and CRSI perform at least as well as CURB-65 or CRB-65, respectively. The analyses suggest a trend of CURSI and CURASI both showing higher sensitivity with only a slight reduction in specificity, and similar results were seen when comparing CRSI and CRASI to CRB-65. These results reflect previous findings in the literature with regard to the higher sensitivity of CURSI/CURASI compared to CURB-65 criteria [20]. A limitation of the CRB-65/CRSI/CRASI analysis was that this was an inpatient cohort rather than outpatients or those seen in the community by general practitioners and therefore may have reduced generalisability to this setting. In primary care, outcomes such as hospital admission, clinical deterioration, or reconsultation may be of as much interest to the general practitioner as mortality or ICU admission.

Replacing increasing age and blood pressure with the shock index may have greater predictive value of mortality and/or admission to ICU. This would be encouraging in the clinical setting as identifying patients who have the greatest need for specialist treatment is of most importance. The unadjusted shock index calculation can be done readily at the bedside simply by sight recognition of whether the heart rate has exceeded the systolic blood pressure. If it has, then the ratio will be greater than 1 and a point can be added to the patient's score. This ease of calculation may also prove beneficial where the age of the patient is unknown due to patient confusion or in the emergency setting.

The data was collected thirteen years ago with the patient cohort managed using hospital guidelines of that time. We used this data for its hypothesis generating ability as it has the thought provoking value that patients were managed and triaged in the absence of CURB-65 criteria because, unlike cohorts in recent studies, this study's cohort predates the derivation and widespread use of CURB-65. Therefore, the results are not confounded by CURB-65, neither, firstly, playing a role in the severity assessment of individual patients nor, secondly, guiding the treatment decisions made during patient care that may have influenced ICU admission or mortality. This allows for the unbiased comparison of each of the scoring criteria against each other, to see what the predicted outcomes would be if any of the three criteria had actually been applied at the time of admission.

It is of note that, with a median age of 61 years, the findings are in a younger cohort than the original CURB-65 derivation study (median age 69) [5]. While death is generally a rare occurrence in previously healthy younger adults, most studies have validated CURB-65 based on the risk of death in older cohorts, so overall risk to younger patients remains relatively unclear. One study did show that CURB-65 had a low sensitivity (54%) for predicting need for mechanical ventilation and/or inotropic support in patients less than 50 years of age [22]. The current study adds to this result by suggesting that CURB-65 has lower sensitivity than shock index criteria for predicting the need for intensive treatment in a relatively young cohort of patients. We did not adjust for the effect of drugs that patients were already taking which may have had an impact on the heart rate and blood pressure, for example, beta-blockers. However, the study purpose was to examine the usefulness of simple clinical parameters to help clinical decision-making (e.g., urea level does not need to take into consideration kidney function in CURB-65).

The application of age-independent criteria, such as CURSI and CURASI, may offer promising alternative severity indices, by removing the possibility of false negatives on the basis of younger age. The inclusion of the shock index may also address concerns over CURB-65 predictions in older patients whose increasing prevalence of systolic hypertension may interfere with severity scoring. Equally, in the absence of urea measurements, calculating the shock index could provide a useful and practical predictive tool in both primary care or emergency settings and help guide the need for admission and acceleration of treatment. The CRSI and CRASI also offer criteria of interest in this regard.

## 5. Conclusions

Given the high mortality still associated with community-acquired pneumonia, there are continued purpose and benefit to find new means of assessing patients. Our hypothesis generating study has findings suggesting that CURSI/CURASI and CRSI/CRASI show potential in severity assessment. Further prospective studies with larger cohorts and wider age ranges in different populations and settings are required.

## 6. Ethical Approval

The study was conducted as part of an internal audit cycle to assess the management and outcome of community-acquired pneumonia. Local ethical approval is not required for audits in the UK.

## Figures and Tables

**Table 1:** Sample characteristics for selected variables included in CURB-65, CURSI, and CURASI criteria.

Variables	median	(range)/number (%)	mean (±sd)
Age (all)	61	(17–96)	58.8 (±18.752)
Age group			
<65 years	51	(53.6%)	
≥65 years	44	(46.3%)	
Sex			
Men	54	(56.8%)	
Women	41	(43.1%)	
Confusion	27	(28.4%)	
Urea	6.65	(1–30.9)	7.9 (±5.111)
Resp. rate	24	(10–40)	24.2 (±6.455)
Blood pressure			
Systolic	130	(75–208)	130.8 (±26.995)
Diastolic	74	(40–113)	72.9 (±14.409)
Pulse rate	100	(56–170)	101.86 (±19.38)
Temperature	37.9	(33.4–40)	37.78 (±1.1756)

Data presented as median (range)/mean (±sd) for continuous data or number (%) for categorical data.

**Table 2:** Sensitivity, specificity, positive and negative predictive values, and corresponding 95% confidence intervals for CURB-65, CURSI, and CURASI criteria for six-week mortality and for mortality and/or ICU admission in 95 community-acquired pneumonia cases.

All (*n* = 95)	Mortality			Mortality and/or ICU admission		
	CURB-65	CURSI	CURASI	CURB-65	CURSI	CURASI
Sensitivity	87.5% (46.7–99.3)	87.5% (46.7–99.3)	87.5% (46.7–99.3)	72.7% (39.3–92.6)	81.8% (47.7–96.8)	81.8% (47.7–96.8)
Specificity	75.8% (65.3–84.1)	72.4% (61.6–81.1)	72.4% (61.6–81.1)	76.2% (65.4–84.5)	73.8% (62.9–82.5)	73.8% (62.9–82.5)
Positive predictive value	25.0% (11.4–45.2)	22.6% (10.3–41.5)	22.6% (10.3–41.5)	28.6% (13.9–48.8)	29.0% (14.9–48.2)	29.0% (14.9–48.2)
Negative predictive value	98.5% (90.9–99.9)	98.4% (90.5–99.9)	98.4% (90.5–99.9)	95.5% (86.6–98.8)	96.9% (88.2–99.4)	96.9% (88.2–99.4)

**Table 3:** Sensitivity, specificity, positive and negative predictive value, and corresponding 95% confidence intervals for CRB-65, CRSI, and CRASI criteria for six-week mortality and for mortality and/or ICU admission in 95 community-acquired pneumonia cases.

All (*n* = 95)	Mortality			Mortality and/or ICU admission		
	CRB-65	CRSI	CRASI	CRB-65	CRSI	CRASI
Sensitivity	50.0% (17.4–82.5)	75.0% (35.6–95.5)	75.0% (35.6–95.5)	33.3% (11.3–64.6)	58.3% (28.6–83.5)	58.3% (28.6–83.5)
Specificity	91.9% (83.6–96.4)	83.9% (74.1–90.6)	85.0% (75.4–91.5)	91.6% (82.8–96.2)	84.3% (74.3–91.1)	85.5% (75.7–91.9)
Positive predictive value	36.4% (12.4–68.4)	30% (12.8–54.3)	31.6% (13.5–56.5)	36.4% (12.4–68.4)	35.0% (16.3–59.0)	36.8% (17.2–61.3)
Negative predictive value	95.2% (87.6–98.5)	97.3% (89.8–99.5)	97.4% (89.9–99.5)	90.5% (81.6–95.5)	93.3% (84.5–97.5)	93.4% (84.7–97.5)
